# Novel mumps virus epitopes reveal robust cytotoxic T cell responses after natural infection but not after vaccination

**DOI:** 10.1038/s41598-021-92926-1

**Published:** 2021-07-01

**Authors:** Patricia Kaaijk, Maarten E. Emmelot, Hugo D. Meiring, Cécile A. C. M. van Els, Jelle de Wit

**Affiliations:** 1 Centre for Infectious Disease Control, National Institute for Public Health and the Environment (RIVM), PO box 41, 3720 BA Bilthoven, The Netherlands; 2 Intravacc (Institute for Translational Vaccinology), Bilthoven, The Netherlands

**Keywords:** Immunology, Medical research

## Abstract

Mumps is nowadays re-emerging despite vaccination. The contribution of T cell immunity to protection against mumps has not been clearly defined. Previously, we described a set of 41 peptides that were eluted from human leukocyte antigen (HLA) class I molecules of mumps virus (MuV)-infected cells. Here, we confirmed immunogenicity of five novel HLA-B*07:02- and HLA-A*01:01-restricted MuV T cell epitopes from this set of peptides. High frequencies of T cells against these five MuV epitopes could be detected ex vivo in all tested mumps patients. Moreover, these epitope-specific T cells derived from mumps patients displayed strong cytotoxic activity. In contrast, only marginal T cell responses against these novel MuV epitopes could be detected in recently vaccinated persons, corroborating earlier findings. Identifying which MuV epitopes are dominantly targeted in the mumps-specific CD8^+^ T- response is an important step towards better understanding in the discrepancies between natural infection or vaccination-induced cell-mediated immune protection.

## Introduction

The implementation of routine measles-mumps-rubella (MMR) vaccination has led to a dramatic reduction in incidence of these infectious diseases, however, mumps is re-emerging nowadays^1,2^. Especially young adults that were vaccinated during childhood can be affected by mumps. Waning of vaccine-induced immunity in the absence of natural boosters by circulating mumps virus (MuV) has been suggested as major cause^3–6^. Various studies have shown a decline of antibody levels in time following childhood vaccination^5,7,8^. However, the finding that antibodies alone do not fully correlate with protection against MuV infection indicates that, apart from the humoral response, other immune compartments play a role in the immunity against MuV^6,9,10^. T cell responses against MuV have been detected in mumps cases as well as in vaccinated healthy persons^9–12^. Previously, we showed that strong MuV-specific CD8^+^ T cells dominate the T cell response in mumps patients. In the same study, we showed that the CD8^+^ T cell response in children that were recently vaccinated with the MMR vaccine was considerably less vigorous than after natural infection^9^. Several scientists have emphasized the importance of fully understanding the T cell immune responses to MuV after vaccination, which is needed to be able to develop strategies to improve the quality and durability of vaccine-induced immunity^2,13–15^. For the detailed characterization of the T cell immune response to MuV and the understanding of its role in natural infection or vaccination, the identification of MuV-specific T cell epitopes is indispensable. Furthermore, to be able to investigate the cross-reactive potency of the T cells induced by vaccination towards the circulating strains, data on the T cell response at the epitope level is also crucial.

Recently, a set of 41 unique viral peptides was eluted from HLA class I molecules of MuV-infected cells (typing: HLA-A*01:01, A*02:01, B*07:02, B*40:01, C*03:04, C*07:02) and characterized by mass spectrometry. From this panel, we confirmed the first six HLA-A*02:01-restricted MuV T cell epitopes to be able to induce a T-cell response^16^. To expand our knowledge on the CD8^+^ T cell repertoire of MuV, we here searched for novel T cell epitopes among the earlier identified panel of naturally processed peptides with high predicted binding scores to the two other common HLA-I molecules, HLA-B*07:02 and HLA-A*01:01. We describe five novel HLA-B*07:02- and HLA-A*01:01-restricted MuV epitopes that reveal for the first time robust ongoing cytotoxic T cell responses in all mumps patients tested, with only low frequencies of epitope-specific T cells in recently vaccinated persons.

## Results

**Figure 1:**
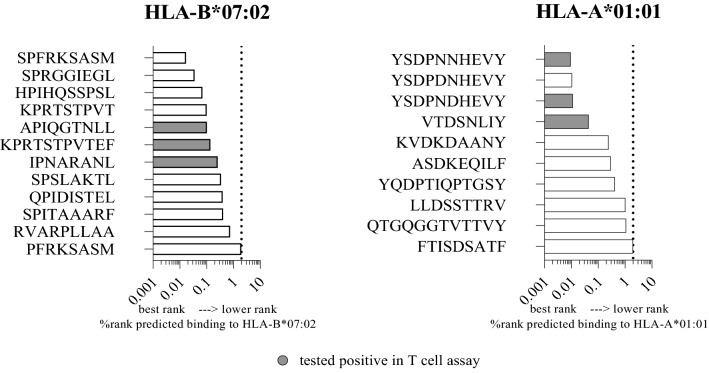
Predicted binding affinity of the candidate epitopes to HLA and capability to induce cytokine production by T cells Mumps virus (MuV) candidate epitopes were selected based on their predicted binding (threshold rank < 2%) to HLA-B*07:02 molecule (left panel; n = 12 peptides) and HLA-A*01:01 molecule (right panel; n = 10 peptides). Selected peptides were used to stimulate peripheral blood mononuclear cells (PBMC) of respectively three HLA-B*0702 positive and three HLA-A*01:01 positive mumps patients. CD8^+^ T effector cell lines from these patients were stimulated with the selected peptides and production of interferon (IFN)-γ and tumor necrosis factor (TNF) by CD8^+^ T cells was measured by flow cytometry. Peptide-reactive T cells were identified as a twofold increase of IFNγ^+^/TNF^+^ cells within the CD3^+^/CD4^-^/CD8^+^ live gate in comparison with medium control. Peptides that were tested positive induced a response in all three HLA-B*07:02 and HLA-A*01:01 positive mumps patients. Filled bars (gray) indicate the peptides that tested positive.

In the present study, peptides from a previously characterized set of 41 naturally processed MuV epitopes^16^, predicted to bind best to HLA-B*07:02 (n = 12) and HLA-A*01:01 molecules (n = 10) were selected for immunogenicity assessment. Selected epitopes were tested for their capacity to recall a specific T cell response, by using expanded CD8 + T-effector cells isolated from three HLA-B*07:02 positive and three HLA-A*01:01 positive mumps patients (Fig. 1).

Three T cell epitopes, i.e., APIQGTNLL (phosphoprotein, aminoacid positions 27–35) V/P/I_27-35_; KPRTSTPVTEF, V/P/I_142-152_ (phosphoprotein); IPNARANL, (nucleoprotein) NP_115-122_, were identified that all elicit a T cell response in 3 out of 3 HLA-B*07:02 positive mumps patients, as measured by IFN-γ and TNF production (Supplementary Fig. 1A). In addition, three HLA-A*01:01-restricted epitopes, i.e., YSDPNNHEVY, (large protein) L_1336-1343_; YSDPNDHEVY, L_1336-1343_; VTDSNLIY, L_1983-1992_, induced a T cell response (Supplementary Fig. 1B). The YSDPNNHEVY peptide, and its deamination variant, YSDPN**D**HEVY (in which the asparagine (N) on position 6 was deaminated to aspartic acid (D)), both induced a T cell response in 3 out of 3 HLA-A*0101 positive mumps patients. The other HLA-A*01:01-restricted epitope (VTDSNLIY) induced IFN-γ and TNF production by CD8^+^ effector T cells from 2 out 3 HLA-A*01:01 positive mumps patients (Fig. 1). Subsequently, APIQGTNLL, KPRTSTPVTEF, IPNARANL (HLA-B*07:02 restricted epitopes), and YSDPNNHEVY and VTDSNLIY (HLA-A*01:01 restricted epitopes) were selected for custom-made (p:HLA) dextramer synthesis to investigate the ex vivo occurrence of CD8^+^ T cells against these epitopes in mumps patients. YSDPNNHEVY and not its post-translationally modified deamination variant, YSDPN**D**HEVY, was selected because it was more abundantly presented based on elution from MHC-I molecules from MuV-infected cells^16^ and had a better predictive binding to HLA-A*01:01 (Fig. 1).

**Figure 2:**
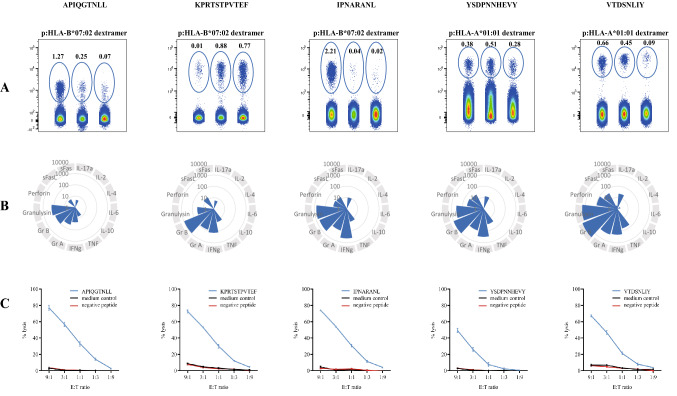
Detection and functional characterization of epitope-specific T cells from mumps patients (**A**) Flow cytometric ex vivo detection of epitope-specific T cells using p:HLA-I dextramers in PBMC samples of three HLA-B*07:02 patients (#03, #08 and #20; shown as respectively the left, middle and right data sets within the first 3 plots of (**A**) and three HLA-A*01:01-positive mumps patients (#04, #14 and #25), shown as respectively the left, middle and right data sets of the last 2 plots of (**A**). Frequencies of p:HLA-class I dextramer-positive cells are presented as percentage of total CD3^+^/CD4^-^/CD8^+^ cells. (**B**) Cytokine analysis of epitope-specific T cell lines derived from HLA-B*07:02- or HLA-A*01:01-positive mumps patients measured with a multiplex bead-based assay quantitating levels of interleukin-2 (IL-2), IL-4, IL-6, IL-10, tumor necrosis factor (TNF), interferon gamma (IFN-γ), granzyme A (GrA), granzyme B (GrB), perforin, soluble Fas ligand (sFasL), soluble Fas (sFas), and granulysin. Concentrations of measured cytokines are all expressed as means of triplicates in pg/mL. (**C**) Cytotoxic activity of epitope-specific T cell lines isolated from HLA-B*07:02- or HLA-A*01:01-positive mumps patients measured with a fl\ow cytometry-based killing assay. Cytotoxic activity of T cells against antigen-presenting cells loaded with the HLA-B*07:02- or HLA-A*01:01-restricted epitopes was measured (blue line). As negative controls, a medium control (black line), as well antigen-presenting cells loaded with a non-immunogenic MuV peptide were included (red line). Specific lysis, presented as mean of triplicates ± SD, was calculated as follows: % specific lysis = [(% dead target cells – % spontaneous dead target cells)/ (100—% spontaneous dead target cells)] × 100% Figures were prepared with following software (**A**) FlowJo V10/Treestar (https://www.flowjo.com/), (**B**) Microsoft 365 Excel (https://www.microsoft.com/en-us/microsoft-365/excel) and (**C**) Graphpad Prism version 9.1.0 (https://www.graphpad.com/scientific-software/prism/).

Using p:HLA dextramers, high frequencies of MuV epitope-specific T cells were observed in all three B*07:02- and three HLA-*01:01-positive patients, even exceeding 2% of the total CD8^+^ T cell pool (Fig. 2A). The frequencies of the epitope-specific CD8^+^ T cells and the dominance of the response per epitope varied between patients. Further analyses showed that the majority of peptide-specific T cells were within the T-effector cell subset (CD3^+^CD8^+^CD45RO^-^CCR7^−^). Functional characterization revealed that, upon stimulation with peptides, the epitope-specific T cells expressed the activation marker CD137 and displayed a cytotoxic phenotype by expression of CD107a. In addition to pro-inflammatory cytokines IFN-γ, TNF and IL-17A, epitope-specific T cells produced granzyme-A/B, granulysin, and to a lesser extent sFas(L), and perforin that are involved in cell-mediated cytotoxicity (Fig. 2B). Finally, we showed that epitope-specific T cells were able to efficiently lyse peptide-pulsed antigen-presenting target cells in a flow cytometry-based cytotoxicity assay (Fig. 2C).

**Figure 3:**
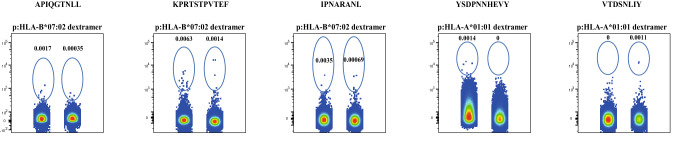
Detection of epitope-specific T cells from recently vaccinated young adults (A) Flow cytometric ex vivo detection of epitope-specific T cells using p:HLA-class I dextramers from PBMC samples of two HLA-B7-positive (#IIV-291-034 (left datasets of first 3 plots) and VAC-076 (right datasets of first 3 plots) to detect responses to three HLA-B*07:02-restricted epitopes, and to detect responses to two HLA-A*01:01-restricted epitopes, PBMC samples of two HLA-A1-positive vaccines were used (#IIV-291-002 (left datasets of last 2 plots) and #025 (right datasets of last 2 plots)). Frequencies of p:HLA-class I dextramer-positive cells are presented as percentage of total CD3+/CD4-/CD8+ cells. Figure was prepared with software FlowJo V10/Treestar (https://www.flowjo.com/).

In contrast to the high frequencies of MuV epitope-specific T cells observed in mumps patients, only very low frequencies of MuV epitope-specific T cells could be detected in young adults that were recently vaccinated with a third dose of the MMR vaccine (Fig. 3). Despite the fact that epitope-specificity varied between individuals, in all tested PBMC samples of HLA-B7- and HLA-A1-positive vaccinated persons, small amounts of MuV epitope-specific CD8^+^ T cells could be visualized with the p:HLA dextramers.

## Discussion

Identification of more functional CD8^+^ T cell epitopes that cover a broad repertoire of T cells against mumps virus (MuV) is important for monitoring and functionally characterizing the T cell response following mumps infection or vaccination. Based on HLA-I binding prediction, most of the set of 41 MuV peptides, from an earlier identified panel^16^, bound well to the HLA-B*07:02 molecule, followed by the HLA-A*02:01 and HLA-A*01:01 molecules, i.e. belonging to the most common HLA‐I alleles^17^. In addition to the previously identified HLA-A*02:01 epitopes, we now confirmed three HLA-B*07:02-restricted T cell epitopes (i.e. APIQGTNLL (V/P/I_27-35_), KPRTSTPVTEF (V/P/I_142-152_), IPNARANL (NP_115-122_)), each inducing a strong T cell response in expanded effector T cells obtained from three different HLA-B*07:02-positive mumps patients. Interestingly, the first described MuV-specific CD4^+^ T-helper cell epitope, GTYRLIPNARANLTA (NP_110-124_), which we recently discovered^18^, contains the newly identified HLA-B*07:02-restricted CD8^+^ T cell epitope IPNARANL (NP_115-122_). This opens the possibility of using synthetic peptides, containing both epitope sequences, to stimulate CD4^+^ and CD8^+^ T cell responses simultaneously. In addition to the HLA-B*07:02-restricted epitopes, HLA-A*01:01-restricted T cell epitopes (i.e. YSDPNNHEVY (L_1336-1343_) and VTDSNLIY (L_1983-1992_)) showed to induce a T cell response in HLA-A*01:01 positive mumps patients. Strikingly, all newly identified HLA-B*07:02- and HLA-A*01:01-restricted T cell epitopes had predicted HLA-I binding ranks < 0.30, i.e. corresponding to the top 0.3% scores of > 850,000 random natural peptides with respect to predicted binding affinity to the HLA-I molecule in question^19^. Thus, in general, the identified immunogenic MuV peptides were predicted to bind more strongly to HLA-I than the non-immunogenic peptides did (Fig. 1), although not all strong HLA-binding peptides appeared to be immunogenic. However, it should be taken into account that expanded CD8 + T-effector cell lines were used for immunogenicity testing of the eluted epitope candidates. It may therefore be that, due to relatively higher frequencies of T cells reactive against the immunodominant T cell epitopes, subdominant epitopes against which only few T cells were reactive could not be not identified; T cells recognizing subdominant epitopes may be overgrown by the more dominant T cell clones represented in higher frequencies.

Four out of the five novel MuV epitopes that were all derived from the genotype G outbreak strain (MuVi/Utrecht.NLD/40.10) appeared to be conserved among the two Jeryl Lynn vaccine strains (JL2 and JL5), and therefore are useful for monitoring cellular immune responses after natural infection with the genotype G outbreak strain and after vaccination. Interestingly, one of the HLA-B*07:02-restricted epitopes, i.e. APIQGTNLL (V/P/I_27-35_) of the genotype G outbreak strain (MuVi/Utrecht.NLD/40.10) differed from the peptide sequences in the two Jeryl Lynn vaccine strains (JL2 (APIQGTN**S**L) and JL5 (**T**PIQGTN**S**L)) and also showed to have sequence differences among various circulating mumps strains, including genotype G, H and C strains. Future experiments are planned to explore the impact of this mismatch on the T cell recognition of the various MuV strains, i.e. vaccine *versus* circulating MuV strains. Using peptide-HLA class I (p:HLA-I) dextramers, high frequencies of T cells against all five MuV epitopes were ex vivo detected in PBMCs of all three HLA-B*07:02- and three HLA-A*01:01-positive mumps patients. Individual differences in epitope dominance of the T cell responses were observed, where one patient had a stronger T cell response to (a) particular epitope(s), another patient showed to have a stronger T cell response to (an)other epitope(s). Functional characterization of these specific T cells revealed a cytotoxic phenotype with expression of degranulation marker CD107a, and secretion of granzyme-A/B, granulysin, perforin, sFas(L). With a flow cytometry-based cytotoxicity assay, epitope-specific T cells showed indeed to be able to efficiently lyse MuV peptide-pulsed target cells. Thus, we for the first time describe that MuV epitope-specific CD8^+^ effector T cells isolated from mumps patients have a cytotoxic phenotype which is a critical feature for mediating viral clearance following acute MuV infection. Although T cells against the novel MuV epitopes were also detectable in recently vaccinated persons, only very low frequencies of these specific CD8^+^ T cells were observed, which is in line with our previous findings^9^. Following respiratory virus infection, a robust CD8 + T cell response can be induced to mediate viral clearance^20^. The question is whether an attenuated virus vaccine strain with lower infection efficiency will be able to induce a comparable strong CD8 + T cell response. Based on our previous findings, where the mumps-specific CD4/CD8 T cell ratio was higher after vaccination than after natural infection, MuV vaccination seems to induce relatively more CD4^+^ than CD8^+^ T cells^9^. Both CD4^+^ and CD8^+^ T cells are involved in protection against viral disease. Prevention of infection can be achieved by induction of CD4^+^ T cells providing help to B cells to produce protective virus-neutralizing antibodies, whereas vaccine-induced CD8^+^ T cells support disease attenuation and protection against complications after infection^21^.

In order to be able to accurately measure the low CD8^+^ T cell response after vaccination, we propose that not a single peptide but rather a pool of confirmed immunogenic peptides representing a broad HLA-I coverage should be used. Such a peptide pool would provide a useful tool to monitor and functionally characterize the CD8^+^ T cell response to MuV to better understand its role after natural infection and vaccination. In addition, MuV epitopes with sequence variations between vaccine and circulating MuV strains can be used to explore the impact of this mismatch on immunity of vaccinated persons to antigenic different circulating MuV strains.

## Methods

### Subjects and blood sample handling

Peripheral blood samples of mumps patients (19–40 years) used for this study were collected in 2012, during a mumps epidemic (genotype G) that spread across multiple locations within the Netherlands. Sampling of the mumps patients took place 1–2 months after disease onset as part of the previously described clinical study VAC-263 (NL37852.094)^22^. In addition, peripheral blood samples were used that were collected from young adults (18–30 years) four weeks after they received a third dose of the MMR vaccine as part of our MMR3 clinical study^6^ or from our blood donor system^23^. Written informed consent was obtained for all participants. The clinical studies were approved by the medical ethical committee (METC Noord-Holland) and performed in accordance with the Declaration of Helsinki.

HLA class I typing (B*07:02 and HLA-A*01:01) was performed by high resolution next-generation sequencing (University Medical Center Utrecht, the Netherlands) or by flow cytometry using anti-HLA class I A1-biotin antibody (8.L.104, , AB31563, Abcam) with streptavidin PE-CF594 (562,318, BD) and anti-HLA class I B7-PE antibody (BB7.1, 372,403, Biolegend) utilizing LSRFortessaX20 (BD) and analyzed with FlowJo (V10/Treestar). Peripheral blood mononuclear cells (PBMCs) were isolated from the blood samples by density gradient centrifugation according to manufacturer's instruction ((Lymphoprep, 1,114,547, Progen) or Cell Preparation Tubes (CPT, BD))and cryopreserved at − 135 °C before thawing for use. Epstein-Barr virus-transformed B-lymphoblastoid cell lines (BLCL) as antigen-presenting cells, were generated from PBMCs as described before^23^.

### Prediction of binding of MuV peptides to HLA-A*01:01 and B*07:02 molecules

The 41 MuV peptides, that were previously eluted from HLA class I molecules of antigen-presenting cells that were infected with the genotype G outbreak strain (MuVi/Utrecht.NLD/40.10)^16^, were tested for predicted binding to the (common) HLA-A*01:01 and B*07:02 molecules using the NetMHCpan 4.0 server. A threshold for selection of best-binding peptides was set on rank < 2%^19^.

### Generation of effector CD8^+^ T cell lines

In order to generate effector CD8^+^ T cell lines, PBMCs from three HLA-B*07:02 positive and three HLA-A*01:01 positive mumps patients were thawed. Cells were stained with fluorochrome-conjugated antibodies anti-CD3-APC/R700 (659,119, BD), anti-CD4-BV711 (563,028, BD), anti-CD8-BV786 (563,823, BD), anti-CD45RO-PE/Cy7 (560,608, BD), and CCR7-PerCP/Cy5.5 (B286436, BioLegend). A subset of the effector T cells (CD3^+^, CD8^+^, CCR7^−^, CD45RO^−^) was sorted using FACSMelody sorter (BD) in order to increase MuV-specific T cell frequencies for peptide immunogenicity testing. Sorted T cells were further expanded in AIM-V medium (12,055–083, Gibco) supplemented with 2% human AB serum (H6914, Sigma), with irradiated (3000 rad) allogeneic PBMCs and irradiated (5000 rad) allogeneic BLCL cells, 1 μg/ml phytohemagglutinin (L4144, Sigma), and 5 ng/ml IL-2 (130–097-743, Miltenyi Biotec). Every 3–4 days IL-2 (5 ng/ml) was added. Cells were frozen when they reached a total of ≥ 2 × 10^6^.

### Immunogenicity testing for CD8^+^ T cell epitope identification

Thawed CD8^+^ T-effector cell lines were cultured for 6 h in AIM-V containing 2% human AB serum in the presence of one of the selected best-binding peptides (1 μM; synthesized by JPT Peptide Technologies Inc). In addition, CD8^+^ T-effector cell lines cultured with medium only (negative control) or in the presence of anti-CD3/CD28 Human T-Activator Dynabeads (11131D, Thermo Fisher Scientific) (positive control) were included. During the last 4 h, Brefeldin (555,029, BD) A and Monensin (554,724, BD) were added. Stimulated cells were stained with fluorochrome-conjugated antibodies, anti-CD3-BUV395 (563,546, BD), anti-CD4-BV711, anti-CD8-BV786, and Fixable Viability Stain 780 (565,388, BD). For intracellular staining, cells were fixed, permeabilized and stained with anti-IFNγ-APC (554,702, BD) and anti-TNF-FITC (2,204,971, Thermo Fisher Scientific), using the Foxp3/transcription factor staining buffer set (00–5523-00, Thermo Fisher Scientific). Cells were acquired on an LSRFortessaX20 (BD) and analyzed using FlowJo (V10/Treestar).

### Ex vivo detection of MuV epitope-specific CD8^+^ T cells from mumps patients and vaccinees

Five immunogenic peptides were selected that were able to induce a T cell response. Subsequently, fluorochrome-labeled peptide-HLA class I (p:HLA-I-)dextramers each loaded with one of these five peptides were synthesized: B*0702-APIQGTNLL (FITC-labeled), B*0702-KPRTSTPVTEF (PE-labeled), B*0702-IPNARANL (APC-labeled), A*0101-YSDPNNHEVY (PE-labeled), and A*0101-VTDSNLIY (APC-labeled) (Custom made, all Immudex). In order to detect ex vivo MuV-specific CD8^+^ T cells, 4 × 10^6^ PBMCs from three HLA-B*0702- and three HLA-A*0101-positive mumps patients and from two HLA-B7- and two HLA-A1-positive young adults that recently received a third dose of the MMR vaccine, were stained with a combination of the different p:HLA-I-dextramers and incubated for 10 min at room temperature. Subsequently, cells were stained with anti-CD3- APC/R700, anti-CD4-BUV737 (564,305 ,BD), anti-CD8-BV786, anti-CD45RO-BUV395 (564,291, BD) and anti-CCR7 for 15 min at 4 °C. On average 175,000–350,000 CD8^+^ T cells were acquired per sample on an LSRFortessaX20.

### Expansion of MuV epitope-specific CD8^+^ T cells from mumps patients

Epitope-specific CD8^+^ T cell lines were generated by sorting dextramer-positive cells (FACSMelody sorter (BD)) from HLA-B*0702- and HLA-A*0101-positive mumps patients and subsequently further expanded as described above for effector CD8 + T cell lines.

### Functional characterization of epitope-specific CD8^+^ T cells

#### Cytokine secretion analysis

Cytokine release of epitope-specific T cell lines was measured after stimulation with one of the five selected peptides (5 µM) or medium as negative control, and performed in triplicate. Cell-free culture supernatant was collected to determine levels of IL-2, IL-4, IL-6, IL-10, IL-17A, TNF, IFN-γ, granzyme A (GrA), granzyme B (GrB), perforin, soluble Fas ligand (sFasL), soluble Fas (sFas), and granulysin in a multiplex bead-based assay (LEGENDplex human CD8/NK panel, 740,267; BioLegend) according to the manufacturers’ instructions and using FACSCantoII (BD).

#### Testing for expression of functional markers

Epitope-specific cell lines were stained with anti-CD3, anti-CD4, anti-CD8, anti-CD45RO and anti-CCR7 for 15 min at 4 °C. Anti-CD107a-BV421 (328,626, BioLegend) was added during culture. For intracellular staining, cells were fixed, permeabilized, and stained with anti-CD137-PE (555,956, BD), anti-IFN-γ and anti-TNF using the Foxp3/transcription factor staining buffer set. Cells were acquired on an LSRFortessaX20.

#### Testing for cytotoxicity capacity

A flow cytometry-based killing assay was used to determine the cytotoxic capacity of the various epitope-specific T cell lines^18^. For this purpose, BLCL as antigen-presenting cells were labeled with 0.1 µM CellTrace violet (C34557, Thermo Fisher Scientific) and pulsed with one of the five selected peptides (5 µM) or medium or non-immunogenic MuV peptide as negative control. Subsequently, epitope-specific T cell lines and BLCL were co-cultured at different effector/target (E:T) ratios in AIM-V supplemented with 2% human AB serum. After 4 h, cells were stained with Fixable Viability Stain 780 (BD) and fixated. Cells were acquired using a LSRFortessa X20. Measurements were made in triplicate and averaged.

## Supplementary Information

Supplementary Information.

## Data Availability

NetMHCpan-4.0 server^19^ was used to predict binding of the set of 41 mumps virus (MuV) peptides to the HLA-A*01:01 and B*07:02 molecules. This server is available via https://services.healthtech.dtu.dk/service.php?NetMHC-4.0
