# Supplementary material for: Novel mumps virus epitopes reveal robust cytotoxic T cell responses after natural infection but not after vaccination
Source: Sci Rep. 2021 Jul 1;11:13664. doi: 10.1038/s41598-021-92926-1 (PMC8249633; doi:10.1038/s41598-021-92926-1)

SUPPLEMENTARY FIGURE 1

Novel mumps virus epitopes reveal robust cytotoxic T cell responses after natural infection but not after vaccination

Patricia Kaaijk[1*], Maarten E. Emmelot[1], Hugo D. Meiring[2], Cécile A.C.M. van Els[1], Jelle de Wit[1]

[1]*Centre for Infectious Disease Control, National Institute for Public Health and the Environment, Bilthoven, The Netherlands*
[2]*Intravacc (Institute for Translational Vaccinology), Bilthoven, The Netherlands*

Supplementary Figure 1. Immunogenicity testing for CD8+ T cell candidate epitopes

The 22 peptides, that were selected based on their predicted binding to HLA-B*07:02 (n=12) and HLA-A*01:01 (n=10) molecules, were tested for their ability to stimulate CD8+ T effector cell lines to produce of interferon (IFN)-γ and tumor necrosis factor (TNF). Flow cytometric detection of IFN-γ and TNF produced by
(A) CD8+ T effector cell line derived from HLA-B*07:02-positive mumps patient (patient #08) upon stimulation with the 12 selected HLA-B*07:02-restricted peptides,
(B) or by (B) CD8+ T effector cell line derived from HLA-A*01:01-positive mumps patient (patient #15) upon stimulation with 10 selected HLA-A*01:01-restricted peptides.
In addition, T cell lines were stimulated with medium (negative control) and anti-CD3/CD28 beads (positive control).
Responding peptide-reactive T cells were identified as a 2-fold increase of IFNγ+/TNF+ cells within the CD3+/CD4-/CD8+ live gate in comparison with medium control (indicated with red line around square).

Kaaijk, *et al.* (2021) "Novel mumps virus epitopes reveal robust cytotoxic T cell responses after natural infection but not after vaccination"

Supplemental Figure 1. Immunogenicity testing for CD8+ T cell candidate epitopes

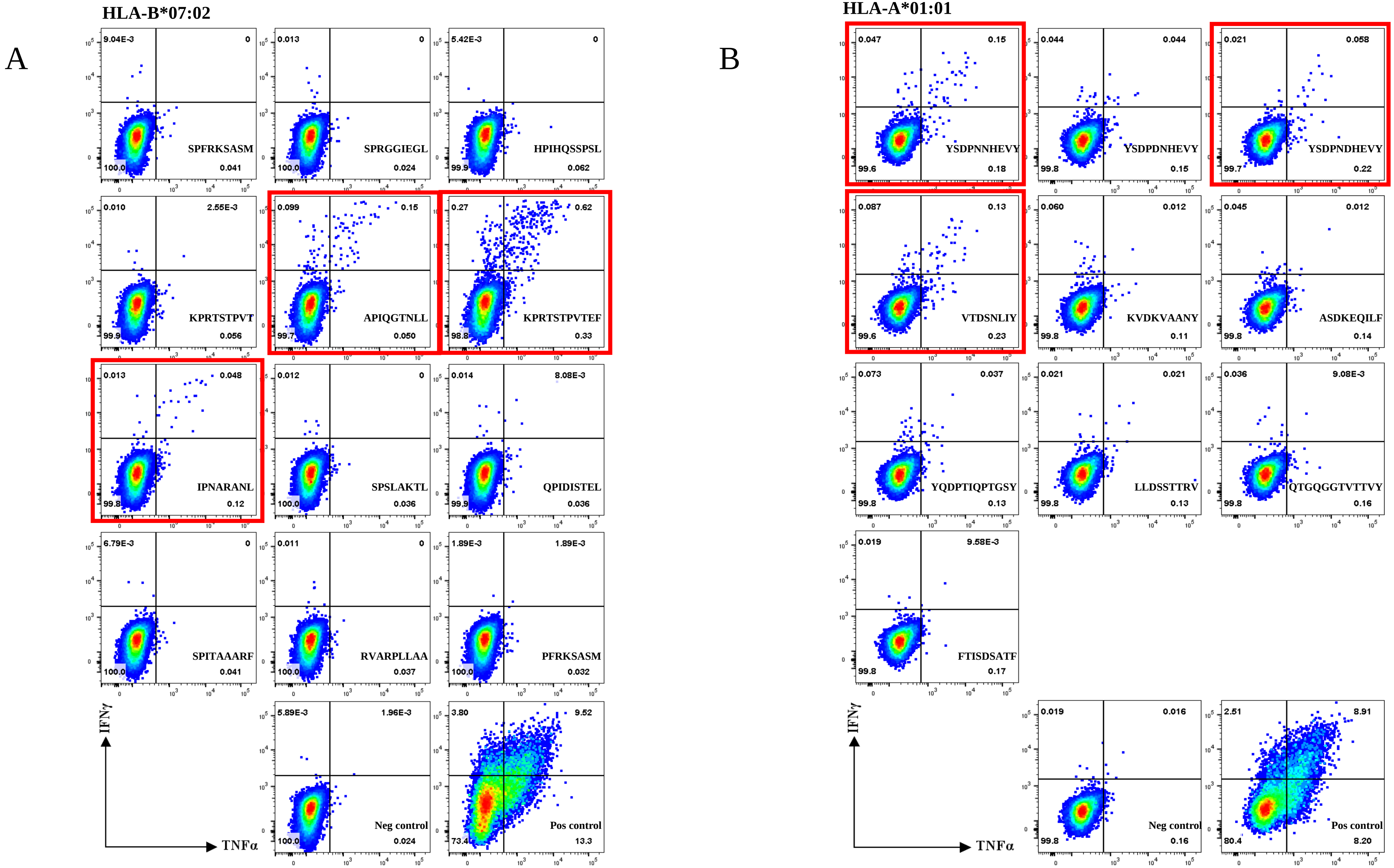

Supplement: Supplementary file 1 — Supplementary Information. [file 41598_2021_92926_MOESM1_ESM.pdf]
